# Stent Selection in Preoperative Biliary Drainage for Patients With Operable Pancreatic Cancer Receiving Neoadjuvant Therapy: A Meta-Analysis and Systematic Review

**DOI:** 10.3389/fsurg.2022.875504

**Published:** 2022-06-30

**Authors:** Jianbing Du, Xiangyu Gao, Hongtao Zhang, Zhuo Wan, Hengchao Yu, Desheng Wang

**Affiliations:** ^1^ Department of Hepatobiliary Surgery, Xijing Hospital, Fourth Military Medical University, Xi’an, China; ^2^ Department of Neurosurgery, Xijing Hospital, Fourth Military Medical University, Xi’an, China; ^3^ Department of Hematology, Tangdu Hospital, Fourth Military Medical University, Xi’an, China

**Keywords:** malignant biliary obstruction, pancreatic cancer, preoperative biliary drainage, metal stent, plastic stent, neoadjuvant therapy

## Abstract

With the increasing use of neoadjuvant therapy (NAT) in patients with pancreatic cancer to reduce tumor burden on prognosis, preoperative biliary drainage (PBD) is becoming increasingly necessary. The aim of this study was to summarize the latest evidence and compare the clinical efficacy of metal stents (MS) and plastic stents (PS) in patients undergoing neoadjuvant therapy for operable pancreatic cancer. Eligible studies were searched in PubMed, Embase and Cochrane Library from their inception to September 2021. In this study, RevMan 5.4 was used to perform the analyses. Two randomized controlled trials (RCTs) and six retrospective studies with 316 patients were included. All patients had pancreatic cancer and received NAT before surgical resection. Meta-analysis showed that the rate of endoscopic reintervention in MS (26/143, 18%) group was lower than that of PS (122/153, 80%) group (*P* < 0.05). The rate of stent-related complications in MS group was lower (18/118, 15%) than that of PS (52/117, 44%) group (*P* = 0.02). But there were no significant differences in operative time, operative blood loss, overall postoperative complications, postoperative hospitalization days and total medical costs between the two groups. For operable pancreatic cancer patients undergoing NAT surgery, MS was preferred over PS in terms of the incidence of endoscopic reintervention and stent-related complications. More clinical trials are needed in the future to confirm these data with higher levels of evidence.

## Introduction

Although diagnosis and treatment of pancreatic cancer have improved, the prognosis remains poor (1). Curative resection is the optical treatment strategy for these patients (2, 3). In recent years, the application of NAT in resectable pancreatic cancer has attracted much attention, focusing on improving prognosis (4–6). NAT refers to neoadjuvant chemotherapy or neoadjuvant chemoradiotherapy for patients with pancreatic cancer before pancreaticoduodenectomy, with the purpose of reducing tumor volume and improving surgical treatment effect. The treatment time from NAT initiation to surgical excision is 2–6 months (7, 8). During this period, PBD can be used to treat malignant biliary obstruction caused by pancreatic cancer. Some studies suggest that PBD should not be routinely used compared to earlier surgery (9, 10). However, reducing serum total bilirubin levels to relieve biliary obstruction during NAT was critical for patient safety (1).

Endoscopic stents are commonly used in the treatment of PBD, and there are two stent types: the metal and plastic stents (11). Studies have shown that MS is the standard stent for patients with unresectable pancreatic cancer because it has a longer patency period and fewer complications than PS (12). However, there is no standard opinion regarding stents used in PBD for patients with operable pancreatic cancer undergoing NAT.

Although there have been many studies comparing the use of plastic and metal stents in patients with pancreatic cancer (1, 13–19), there has been no meta-analysis to evaluate patients expected to undergo surgical removal after NAT. The purpose of this systematic review and meta-analysis was to compare the rate of endoscopic reintervention, stent-related complications, postoperative surgical complications, postoperative hospital stay, and total medical costs of metal versus plastic stents for PBD in patients with operable pancreatic cancer undergoing NAT therapy.

## Methods

The present study was carried out according to the Preferred Reporting Items for Systematic Reviews and Meta-Analyses (PRISMA) guidelines (20).

### Literature Search

The comprehensive electronic search was conducted in the PubMed, Embase and Cochrane Library from their inception to September 2021. Fully covered self-expandable metal stent (FCSEMS) and uncovered metal stents were considered. The following search strategy was used in PubMed: ((plastic stent[Title/Abstract]) AND ((((Self Expandable Metallic Stent[Title/Abstract]) OR (Self Expandable Metal Stent[Title/Abstract])) OR (Self Expandable Metal Stents[Title/Abstract])) OR (metal stent[Title/Abstract]))) AND((((((((((((((((((Neoplasm, Pancreatic[Title/Abstract]) OR (Pancreatic Neoplasm[Title/Abstract])) OR (Pancreas Neoplasms[Title/Abstract])) OR (Neoplasm, Pancreas[Title/Abstract])) OR (Neoplasms, Pancreas[Title/Abstract])) OR (Pancreas Neoplasm[Title/Abstract])) OR (Neoplasms, Pancreatic[Title/Abstract])) OR (Cancer of Pancreas[Title/Abstract])) OR (Pancreas Cancers[Title/Abstract])) OR (Pancreas Cancer[Title/Abstract])) OR (Cancer, Pancreas[Title/Abstract])) OR (Cancers, Pancreas[Title/Abstract])) OR (Pancreatic Cancer[Title/Abstract])) OR (Cancer, Pancreatic[Title/Abstract])) OR (Cancers, Pancreatic[Title/Abstract])) OR (Pancreatic Cancers[Title/Abstract])) OR (Cancer of the Pancreas[Title/Abstract])) OR (Pancreas Neoplasms[MeSH Terms])). Similar processes have been performed in the Cochrane Library and Embase databases to expand data retrieval. The data were extracted by investigators and any discrepancies were resolved by re-evaluating the data.

### Inclusion and Exclusion Criteria

The inclusion criteria were: (1) studies should focus on metal versus plastic stents for PBD in patients with resectable and borderline resectable pancreatic cancer; (2) patients receiving neoadjuvant therapy before surgical resection; (3) the study showed at least stent-related and postoperative adverse events. The exclusion criteria were: (1) patients with unresectable pancreatic cancer; (2) the article was review, protocol and case report; (3) studies with incomplete information. In this study, stent-related complications included hemorrhage after PBD, acute pancreatitis, acute cholecystitis, cholangitis and perforation; postoperative complications included hemorrhage after surgery, pancreaticojejunostomy leakage, biliary leakage, acute hepatic failure, delayed gastric emptying, acute renal failure, wound infection, portal vein thrombosis and pneumonia.

### Study Quality and Risk of Bias Assessment

The quality of enrolled cohort studies was assessed by a quality checklist recommended by the Cochrane Handbook (21). Two investigators conducted independent quality assessment and reached a consensus after discussion.

### Statistical Analysis

RevMan software version 5.4 (The Nordic Cochrane Center, Cochrane Collaboration, Copenhagen, Denmark) was used in the meta-analysis. For dichotomous variables, the odds ratio (OR) with 95% confidence interval (CI) was calculated to compare the incidence of stent-related complications, rate of endoscopic reintervention and postoperative surgical complications between the metal and plastic stents groups. For quantitative analysis of continuous variables, the weighted mean differences (WMD) with 95% confidence interval (CI) were used to compared the postoperative hospital stay and total medical costs between the two groups. If the reported data was median (range), we recalculated the estimated sample mean and standard deviation (SD) based on the trial data according to the reported calculation method (22, 23). Heterogeneity was qualitatively evaluated using a χ^2^-based Q test (chi-square test). The level of heterogeneity was evaluated by Ι^2^ statistics. If Ι^2^ < 50%, it was considered to be low heterogeneity and a fixed effects model was applied. If Ι^2^ > 50%, it represented high heterogeneity and a random effects model was applied. *P* value <0.05 was considered statistically significant. U.S. dollars were used as the unit of the cost, and the exchange rate was 1 JPY = 0.008824 USD in this study.

## Results

### Search Results and Study Characteristics

**Figure 1:**
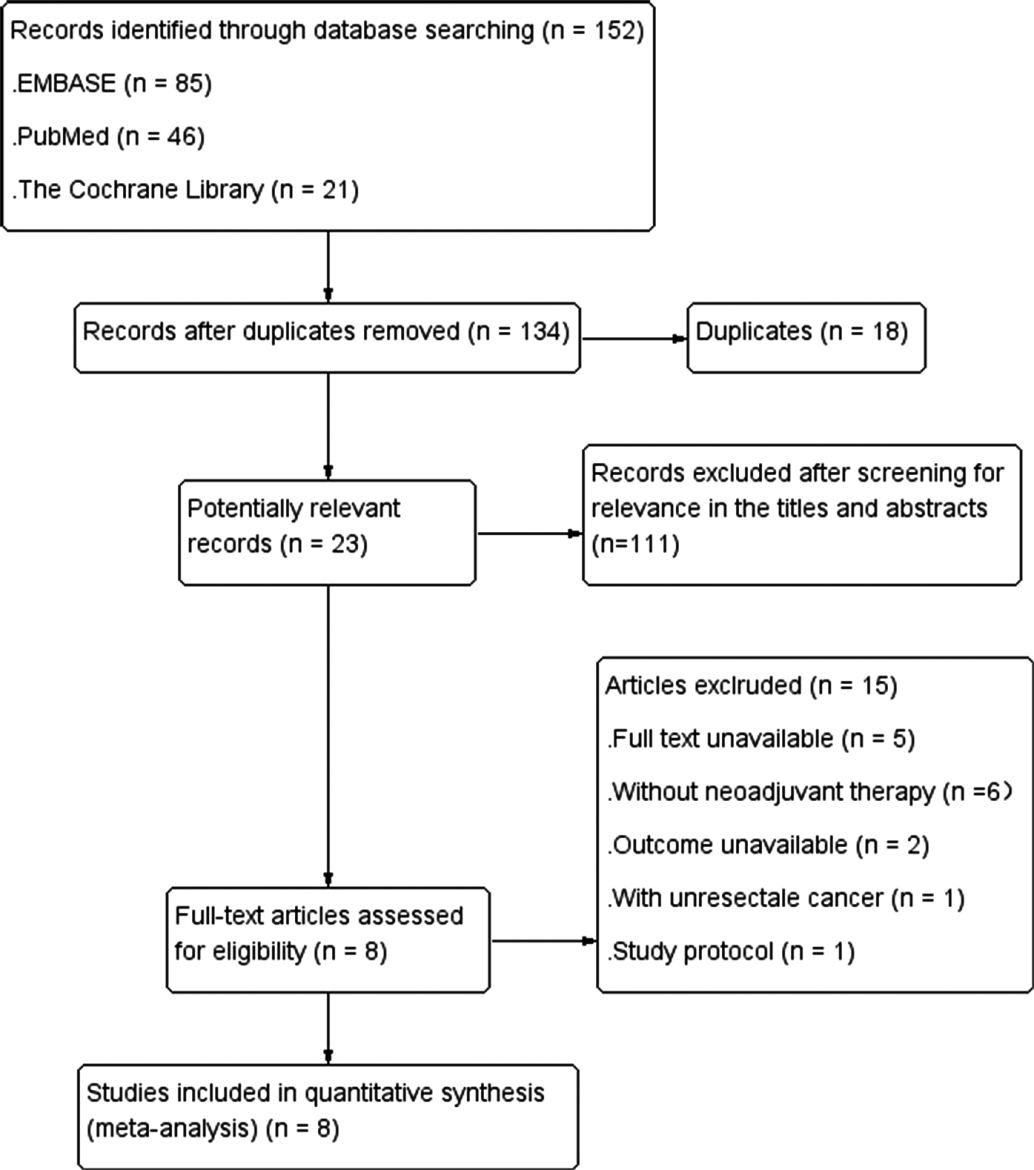
Flow diagram of the process of including and excluding studies for this meta-analysis.

Study selection process was shown in Figure 1. A total of 152 articles were retrieved by searching electronic databases. After 18 duplicates were excluded, 134 articles remained. Then, the titles and abstracts were screened for relevance and evaluated for eligibility, and 111 articles were excluded, leaving 23 articles. The full text of the literatures was obtained. After screening relevant data in the 23 articles, 15 irrelevant articles were excluded, and finally 8 articles were included in our research.

**Figure 2:**
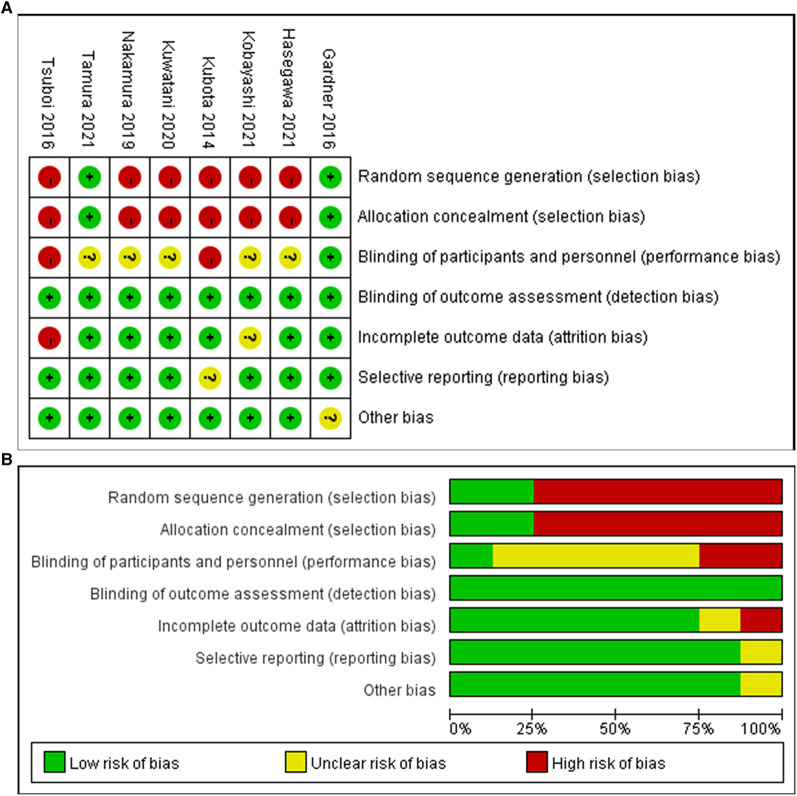
Risk bias diagram of the included studies (**A**) Risk of bias summary; (**B**) Risk of bias graph.

Two of the eight studies (13, 18) were randomized controlled trials and six (1, 14–17, 19) were retrospective cohort trials including 316 patients in total (Tables 1 and 2). Of the 316 patients, 152 patients (48%) were treated with metal stents and 164 patients (52%) were treated with plastic stents. These were no differences in age, gender and tumor size. There is a certain heterogeneity in the types of metal stents in different studies. Some continuous variables (preoperative period, operative time, operative blood loss, hospital stay and total medical cost) were provided with median (range), so we need to recalculated the original data into mean and SD according to the method mentioned above. The quality of enrolled studies was assessed by a quality checklist (Figure 2) and each study had a moderate or high quality.

**Table 1:** Characteristics of the eight studies included in the systematic review and meta-analysis.

First author	year	Period	Country	Study design	sample size	Stent	No. of patients	Male/female	Age(years)	Tumor size, mm
Tamura	2021	2017–2020	Japan	RCT	22	metal	11	7/4	66.6 ± 9.0	24.6 ± 6.4
						plastic	11	5/6	67.4 ± 8.0	27.8 ± 9.0
Hasegawa	2021	2013–2019	Japan	Retrospective	67	metal	27	9/18	68 (43–89)	21 (10–50)
						plastic	40	22/18	67 (46–79)	25 (10–40)
Kobayashi	2021	2009–2018	Japan	Retrospective	43	metal	21	11/10	74 (56–83)	20 (13–32)
						plastic	22	12/10	69.5 (43–85)	23 (12–35)
Kuwatani	2020	2013–2016	Japan	Retrospective	28	metal	17	8/9	66 (50–83)	21 (14–40)
						plastic	12	5/7	68 (53–80)	19 (11–28)
Nakamura	2019	2008–2017	Japan	Retrospective	43	metal	17	12/5	70 (52–76)	
						plastic	26	12/14	61 (36–76)	
Gardner	2016	2010–2013	Lebanon	RCT	54	metal	33	20/13	65.9	33.7
						plastic	21	11/10	65.9	34.2
Tsuboi	2016	2010–2014	Japan	Retrospective	20	metal	9	7/2	63	25.5
						plastic	11	5/6	65	27
Kubota	2014	2009–2012	Japan	Retrospective	38	metal	17	13/4	65.9 (55–76)	35 (20–53)
						plastic	21	13/8	65.6 (48–79)	35 (18–45)

*RCT, randomized controlled trial; No., number.*

**Table 2:** Preoperative, postoperative outcomes and cost of the eight studies.

First author	Stent	No. of patients	Preoperative period (days)	Completion of NAT %	Endoscopic reintervention	Stent-related AE	Postoperat-ive AE, %	Operative time (min)	Operative blood loss (mL)	Hospita-lization days	Stenting cost per patient (USD)	Total medical cost (USD)
Tamura	metal	11	102 ± 7	82% (9)	18% (2)	18% (2)	29% (2)	407 ± 55.2	237 ± 140	25 ± 7	6,210 ± 4,727	45,279 ± 10,711
	plastic	11	99 ± 15	73% (8)	73% (8)	64% (7)	33% (3)	443 ± 95.5	446 ± 378	23 ± 6	7,696 ± 6,081	42,699 ± 8,710
Hasegawa	metal	27	201 ± 143	100% (27)	15% (4)	11% (3)	19% (5)	730 ± 32	1,549 ± 347			
	plastic	40	196 ± 104	100% (40)	97% (39)	13% (5)	20% (8)	704 ± 96	1,534 ± 288			
Kobayashi	metal	21	80 ± 21		5% (1)	10% (2)	29% (6)			22 ± 7	5,876	30,834
	plastic	22	78 ± 22		95% (21)	95% (21)	64% (14)			42 ± 36	4,664	37,843
Kuwatani	metal	17		88% (15)	6% (1)	6% (1)	47% (8)	615 ± 56	742 ± 200		4,973	
	plastic	12		83% (10)	83% (10)	0	50% (6)	489 ± 48	636 ± 191		5,700	
Nakamura	metal	17	75 ± 14	100% (17)	24% (4)		47% (8)	389 ± 166	545 ± 282	19 ± 9	5,641	
	plastic	26	80 ± 15	100% (26)	58% (15)		50% (13)	357 ± 102	1,301 ± 1,694	34 ± 38	5,539	
Gardner	metal	33	164 ± 16		30% (10)	18% (6)					3,847	23,834
	plastic	21	148 ± 22		52% (11)	0					116	18,701
Tsuboi	metal	9				0%	29% (3)	420 ± 129	1,390 ± 1,101	20 ± 4		14,000 ± 7,051
	plastic	11				73% (8)	18% (2)	453 ± 77	1,545 ± 716	23 ± 5		20,757 ± 14,464
Kubota	metal	17	126 ± 50		24% (4)		7% (1)	708 ± 171	974 ± 542	30 ± 11		12,907 ± 3,623
	plastic	21	106 ± 33		86% (18)		5% (1)	809 ± 292	897 ± 482	35 ± 18		12,446 ± 5,167

*NAT, neoadjuvant therapy; No., number; AE, adverse event.*

### Endoscopic Reintervention

**Figure 3:**
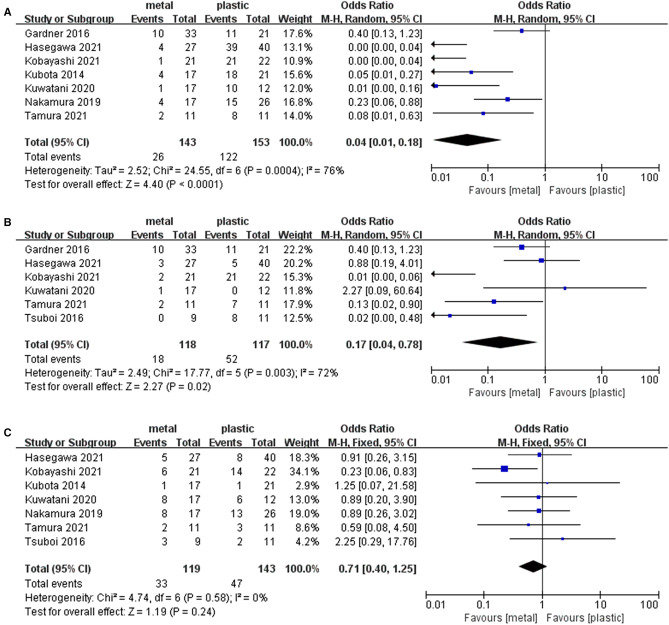
Forest plot of (**A**) Rate of endoscopic reintervention; (**B**) Rate of stent-related complications; (**C**) Rate of overall postoperative complications.

Seven studies (1, 13–16, 18, 19) including 143 cases in metal stents group and 153 cases in plastic group, provided the data about the endoscopic reintervention during PBD. Reintervention was performed when the stent was malfunctioning (worsening jaundice, cholangitis or liver function tests) due to stent migration or stent occlusion (13, 19, 24). Because I^2 ^> 50%, we used a random effects model to analyze the data. The rate of reintervention in metal stents group (26/143, 18%) was significantly lower than that in plastic stents group (122/153, 80%) (OR = 0.04, 95% CI = 0.01–0.18, *P* < 0.0001) (Table 2 and Figure 3A).

### Overall Stent-Related Preoperative Complications

Six studies (13–18) containing 118 cases in metal stents group and 117 cases in plastic group, provided stent-related preoperative complications. Ι^2^ = 72%, *P* = 0.003, a random effects model was used to pool the OR, and the rate of stent-related preoperative complications in metal stents group (18/118, 15%) was statistically lower than that in plastic stents group (52/117, 44%) (OR = 0.17, 95% CI = 0.04–0.78, *P* = 0.02) (Table 2 and Figure 3B).

### Overall Postoperative Complications

Seven studies (1, 13–17, 19) including 119 cases in metal stents group and 143 cases in plastic group, provided postoperative complications. Ι^2^ = 0%, *P* = 0.58, a fixed effects model was used to pool the OR, and the rate of overall postoperative complications in metal stents group was lower (33/119, 28%) than that in plastic stents group (47/143, 33%), but the difference was not statistically significant (OR = 0.71, 95% CI = 0.40–1.25, *P* = 0.24) (Table 2 and Figure 3C).

### Operative Time

**Figure 4:**
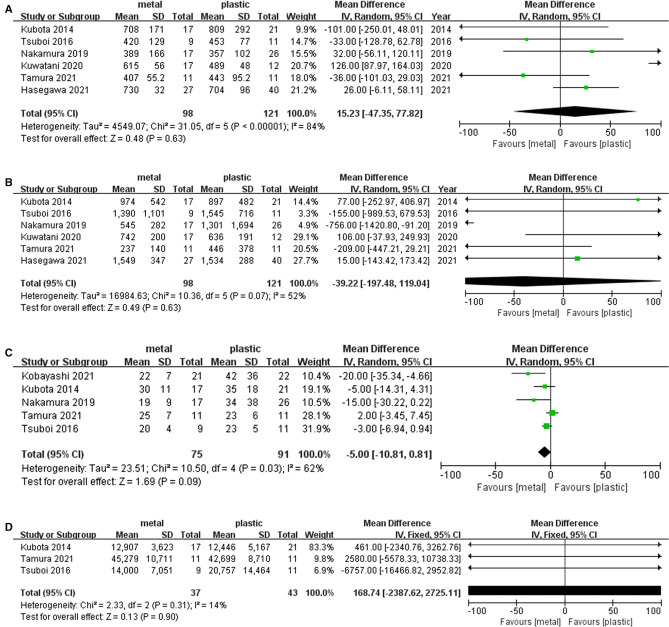
Forest plot of (**A**) Operative time; (**B**) Operative blood loss; (**C**) Postoperative hospitalization days; (**D**) Total medical cost.

Six studies (1, 13, 15–17, 19) including 98 cases in metal stents group and 121 cases in plastic group, provided the data about this outcome. Ι^2^ = 84%, *P* < 0.001, a random effects model was used to pool the mean difference (MD), and the results showed no significant difference between the two groups. (MD = 15.23, 95% CI = −47.23–77.82, *P* = 0.63) (Table 2 and Figure 4A).

### Operative Blood Loss

Six studies (1, 13, 15–17, 19) including 98 cases in metal stents group and 121 cases in plastic group, provided this outcome. Ι^2^ = 52%, *P* = 0.07, a random effects model was used to pool the mean difference (MD), and the results were not statistically significant. (MD = −39.22, 95% CI = −197.48–119.04, *P* = 0.63) (Table 2 and Figure 4B).

### Postoperative Hospitalization Days

Five studies (1, 13, 14, 17, 19) including 75 cases in metal stents group and 91 cases in plastic group, provided this outcome. Ι^2^ = 62%, *P* = 0.03, a random effects model was used to pool the mean difference (MD), and the results were not statistically significant in postoperative hospitalization days. (MD = −5.00, 95% CI = −10.81–0.81, *P* = 0.09) (Table 2 and Figure 4C).

### Total Medical Cost

Five studies provided the data concerning this outcome, but only three studies (13, 17, 19) were included in the analysis because two studies missed standard deviation data. Overall, 37 cases in metal stents group and 43 cases in plastic group were included. Ι^2^ = 14%, *P* = 0.31, a fixed effects model was used to pool the mean difference (MD), and the results were not statistically significant (MD = 168.74, 95% CI = −2387.62–2725.11, *P* = 0.90) (Table 2 and Figure 4D).

### Sensitivity Analysis

**Figure 5:**
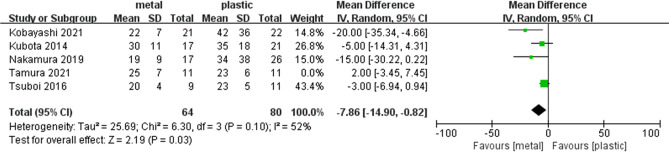
Forest plot of sensitivity analysis of postoperative hospitalization days.

We conducted a sensitivity analysis by omitting one study at a time to assess whether the results may be significantly affected by a single study. Sensitivity analysis showed that the results in the meta-analysis were relatively stable except for postoperative hospitalization days. When omitted Tamura’s trial, the Ι^2^ = 52% and the result was statistically significant. The postoperative hospitalization days was significantly shorter in MS (MD = −7.86, 95% CI = −14.9- (−0.82), *P* = 0.03) (Table 2 and Figure 5).

## Discussion

This is the first systematic review and meta-analysis comparing metal versus plastic stents for preoperative biliary drainage in operable pancreatic cancer patients undergoing neoadjuvant therapy. In our study, we found that the MS group had a lower rate of endoscopic reintervention than the PS group (18% versus 80%, *P *< 0.05). We also found that there was a statistical difference between the two groups in the rate of stent-related complications (15% versus 44%, *P* = 0.02). Furthermore, we found that the cost of the stent in MS group was higher than that in PS group, but there was no significant difference in the total medical costs between the two groups.

In the past, resectable periampullary malignancy were almost always performed with plastic stents for PBD. The main reasons included the high cost of MS and the belief that MS may affect subsequent pancreaticoduodenectomy (25). But in this meta-analysis, it’s not exactly like this. There was no significant difference in PBD in patients with operable pancreatic cancer who received neoadjuvant therapy in terms of surgical outcome. However, the rate of endoscopic reintervention and stent-related complications was lower in MS group than that in PS group. These results were affected by different lumen calibers of MS and PS (12). The diameter of MS was usually larger than PS, so MS could achieve the goal of prolonging the patency of the stent, while PS was more likely to cause occlusion (26, 27). There was no significant difference between the two groups in operation-related indicators (operative time and operative blood loss) and postoperative complications. The main reason is that the clinical experience of surgeon and the complexity of the operation have a great influence on them, while the type of stent may have little influence on them, which requires more clinical data to support. In other words, different types of stents mainly cause different clinical outcome in short-term prognosis rather than long-term effects. In addition, the medical cost is an important factor to consider when treating patients, with a cost-benefit analysis showing that MS cost more per patient than PS (mean costs, MS: $5041; PS: $4371) in Table 2. However, the rate of endoscopic reintervention in PS group was higher than that in MS group, which would lead to increase PBD-related surgeries and thus increase the additional cost burden for patients. More importantly, endoscopic reintervention would increase the patient’s pain and reduce the patient’s quality of life. Therefore, the additional cost of MS was offset by its longer-lasting patency compared with PS. Furthermore, studies have reported that patients receiving NAT are more susceptible to neutropenia caused by chemotherapy, and thus may be more susceptible to infection (14). These patients undergoing NAT would have an increased risk of cholestasis due to the shedding of cellular material produced by chemotherapy, and due to immune damage, bacterial colonization in the stent would also increase, which would eventually lead to an increased risk of stent obstruction and cholangitis (28). These reasons support the superiority of MS over PS in PBD in patients receiving neoadjuvant therapy for operable pancreatic cancer. As for sensitivity analysis, Tamura’s trial was omitted for analysis, statistically different results were obtained, but Tamura’s trial could not be omitted because it was an RCT, which had a relatively high level of evidence. The main reason for the sensitivity analysis results was that the results of Tamura’s trial were inconsistent with other research’s results.

Two randomized controlled trials (RCTs) and six retrospective studies were included in our meta-analysis, which lacked data support from large multi-center clinical trials. Presently, an ongoing study in Korea (NCT04911647) is randomizing between plastic and metal stents in patients receiving neoadjuvant chemotherapy for operable pancreatic cancer. The results of this study are likely to increase the level of evidence on this topic in the future. There were several limitations in this meta-analysis. Firstly, 6 of 8 cohort trials were retrospective design and the sample size was not large enough. Secondly, the included literature lacks detailed data on the types of metal stents, and it was impossible to conduct sub-analysis of different metal stents (the covered and uncovered stents). Thirdly, we estimated the mean and SD based on the data reported as the median (range) for continuous data (for example, operative time, operative blood loss, postoperative hospitalization days and total medical cost), this led to the inability to obtain more accurate data for analysis.

## Conclusion

In summary, this system review and meta-analysis showed that MS was superior than PS for patients with operable pancreatic cancer undergoing neoadjuvant therapy, but there was no significant difference in long-term prognosis between the two groups. However, more clinical trials with higher levels of evidence are needed to confirm these data in the future.

## Data Availability

The original contributions presented in the study are included in the article/Supplementary Material, further inquiries can be directed to the corresponding author/s.
